# Development of a high-throughput fluorescent no-wash sodium influx assay

**DOI:** 10.1371/journal.pone.0213751

**Published:** 2019-03-11

**Authors:** Bryan Tay, Teneale A. Stewart, Felicity M. Davis, Jennifer R. Deuis, Irina Vetter

**Affiliations:** 1 Institute for Molecular Bioscience, The University of Queensland, Brisbane, Queensland, Australia; 2 Mater Research Institute—The University of Queensland, Faculty of medicine, The University of Queensland, Woolloongabba, QLD, Australia; 3 Translational Research Institute, Woolloongabba, QLD, Australia; 4 School of Pharmacy, The University of Queensland, Brisbane, QLD, Australia; University at Buffalo - The State University of New York, UNITED STATES

## Abstract

Voltage-gated sodium channels (Na_V_s) are key therapeutic targets for pain, epilepsy and cardiac arrhythmias. Here we describe the development of a no-wash fluorescent sodium influx assay suitable for high-throughput screening and characterization of novel drug leads. Addition of red-violet food dyes (peak absorbance range 495–575 nm) to assays in HEK293 cells heterologously expressing hNa_V_1.1–1.8 effectively quenched background fluorescence of the sodium indicator dye Asante NaTRIUM Green-2 (ANG-2; peak emission 540 nm), negating the need for a wash step. Ponceau 4R (1 mM) was identified as a suitable quencher, which had no direct effect on Na_V_ channels as assessed by patch-clamp experiments, and did not alter the pharmacology of the Na_V_1.1–1.7 activator veratridine (EC_50_ 10–29 μM) or the Na_V_1.1–1.8 inhibitor tetracaine (IC_50_’s 6–66 μM). In addition, we also identified that the food dyes Ponceau 4R, Brilliant Black BN, Allura Red and Amaranth are effective at quenching the background fluorescence of the calcium indicator dyes fluo-4, fura-2 and fura-5F, identifying them as potential inexpensive alternatives to no-wash calcium ion indicator kits. In summary, we have developed a no-wash fluorescent sodium influx assay suitable for high-throughput screening based on the sodium indicator dye ANG-2 and the quencher Ponceau 4R.

## Introduction

Voltage-gated sodium channels (Na_V_s) are key therapeutic targets for pain, epilepsy and cardiac arrhythmias. The Na_V_ subfamily consists of nine α subunits (Na_V_1.1–1.9), which are responsible for the generation and propagation of action potentials in neurons. The ~260kDa proteins form an ion-selective pore which opens upon membrane depolarization to allow influx of Na^+^ [1]. While patch-clamp electrophysiology remains the gold standard for assessing Na_V_ channel function, fluorescence-based assays are routinely used to screen vast chemical libraries for novel drug leads, as these assays are comparatively cheap, high-throughput and less technically challenging to perform [2]. Unlike patch-clamp electrophysiology, fluorescence-based assays provide an indirect measure of Na_V_ channel function, with many commercial dyes available that detect changes in membrane potential or intracellular ion concentration instead of directly measuring sodium current.

**Fig 1:**
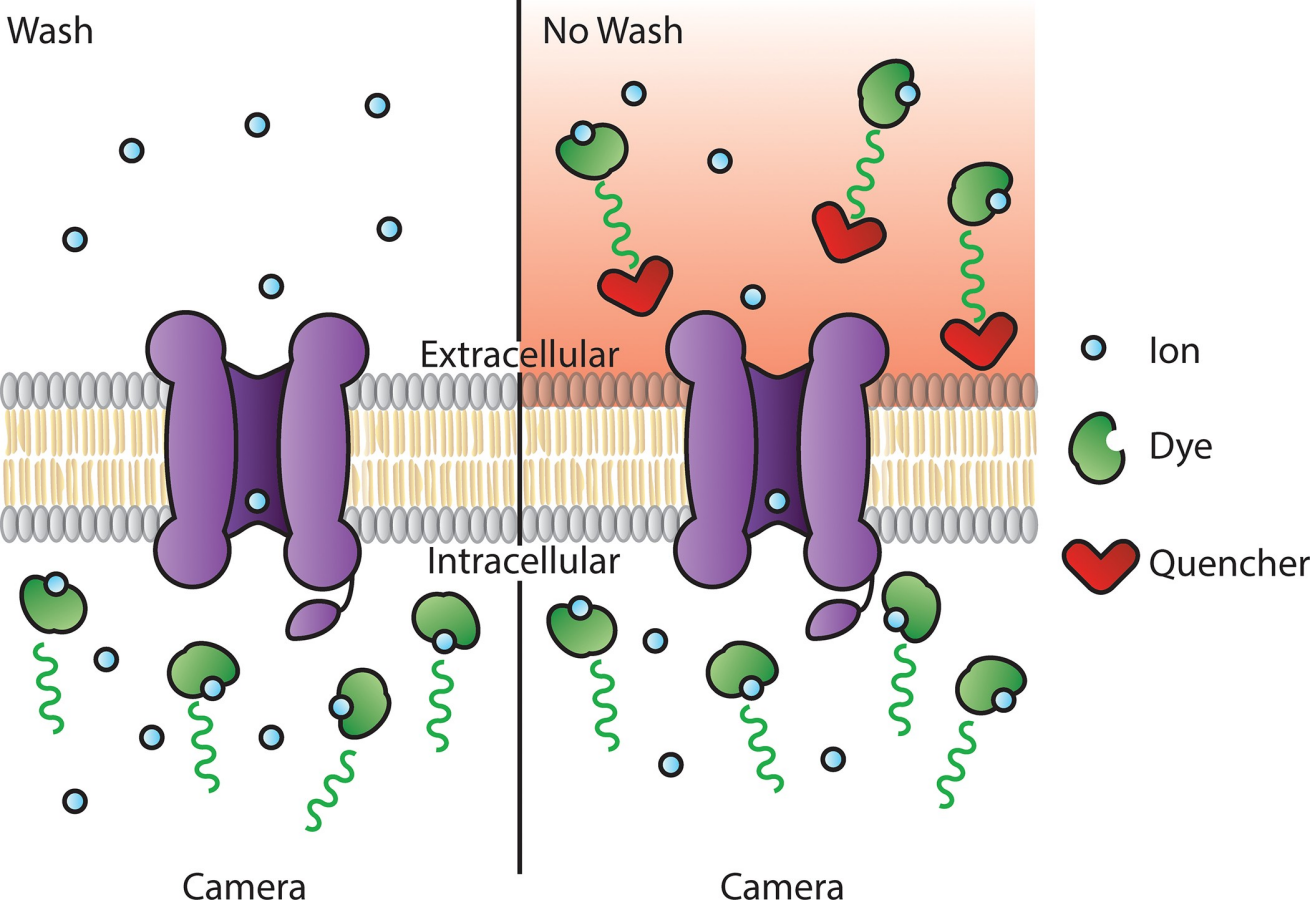
Schematic diagram illustrating the principle of a fluorescent wash (left) and no-wash (right) ion influx assay.

Assays that measure intracellular sodium influx require a sodium indicator dye that emits a fluorescence signal upon binding to sodium ions, with commercial dyes such as SBFI (Sodium-binding benzofuran isophthalate, Thermofisher Scientific), CoroNa Green (Thermofisher Scientific), and Asante NaTRIUM Green-2 (ANG-2, TEFLabs) commercially available. All of these indicator dyes are available as acetoxymethyl (AM) ester-conjugated derivatives, enabling passive diffusion across cell membranes [3]. Once inside the cell, the AM ester is cleaved off by endogenous esterases, causing the dye to be trapped within the cell. In order to isolate intracellular changes in sodium ion concentrations, any extracellular sodium indicator dye needs to be removed by washing (Fig 1). However, a wash step can be difficult to implement in high-throughput formats, as it can cause the detachment of cells with low adherence, lead to well-to-well variability, and increase assay time with an additional step.

An alternate method to reduce background fluorescence is to add an extracellular quencher, which is a membrane-impermeable dye that masks fluorescent signal at the wavelength of the ion indicator dye, negating the need for a wash step [4]. This approach is used in many commercial no-wash assay kits, including the FLIPR Membrane Potential and Calcium Assay Kits (Molecular Devices), however the contents of these kits are usually proprietary and the identity of the quencher is generally not disclosed. An alternative to using commercial no-wash assay kits is to use a food dye as a quencher. The use of food dyes to negate the need for a wash step has been successfully described for thallium and calcium ion influx assays, but not for sodium [5–7].

As there are no commercially available no-wash assay kits for sodium, the aim of this study was to develop and optimize a no-wash sodium influx assay suitable for high-throughput screening of Na_V_ channels using commercially available food dyes as quenchers with the sodium indicator ANG-2 (AM)[8].

## Materials and methods

### Spectrophotometry

Quencher dyes Allura Red AC, Amaranth, Carmine, Brilliant Black BN (Sigma Aldrich), and Ponceau 4R (pillar box red food dye) (Queen Australia) were diluted in water to the maximum concentration required to be within detection range (250–1000 μM). Their absorbance spectra were measured with a NanoDrop^TM^ ND1000 (ThermoFisher Scientific) UV-Vis spectrophotometer and plotted vs UV-Vis spectrum wavelength using GraphPad Prism 6.0.

### Cell culture

HEK293 cells heterologously expressing human Na_V_1.1–1.8 (SB Drug Discovery, Glasgow, UK) were cultured in Minimum Essential Medium (MEM) (Sigma Aldrich #M5650) containing 10% v/v fetal bovine serum (FBS) and selection antibiotics as recommended by the manufacturer. SH-SY5Y human neuroblastoma cells (Victor Diaz, Goettingen, Germany) were cultured in Roswell Park Memorial Institute (RPMI) medium containing 15% v/v FBS and supplemented with L-glutamine (1 mM). MDA-MB-468 breast cancer cells (The Brisbane Breast Bank, UQCCR, Brisbane, Australia) were maintained in Dulbecco’s Modified Eagle’s Medium (DMEM) (Sigma Aldrich, #D6429) containing 10% v/v FBS and supplemented with L-glutamine. Cells were grown in a humidified 5% CO_2_ incubator at 37°C, grown to 70–80% confluence, and passaged every 3–4 days using TrypLE Express (Invitrogen) or trypsin (0.25%).

### FLIPR sodium dye assay

HEK293 cells stably expressing Na_V_1.1–1.8 were plated at a density of 10,000–15,000 cells per well on black-walled 384-well imaging plates and cultured for 48 h. ANG-2 (Abcam) was diluted from a 10 mM stock in DMSO to 10 μM in physiological salt solution (140 mM NaCl, 11.5 mM glucose, 10 mM HEPES, 5.9 mM KCl, 1.4 mM MgCl_2_, 1.2 mM NaH_2_PO_4_, 5 mM NaHCO_3_, 1.8 mM CaCl_2_, pH 7.4) with or without the presence of the quenchers Brilliant Black BN, Carmine, Ponceau 4R, Allura Red and Amaranth at the concentrations stated. Growth media was removed and replaced with ANG-2 solution (20 μL/well) and cells were incubated in the dark at 37°C for 30 min. The change in fluorescence was measured (excitation 470–495 nm, emission 515–575 nm) every 1 s after addition of compounds using the fluorescence imaging plate reader FLIPR^TETRA^ (Molecular Devices) for the durations stated, and analysed using ScreenWorks (Molecular Devices, Version 3.2.0.14). To quantitate the effect of quenching, the average relative light units corresponding to 60–100 s after addition of food dyes was used. To quantitate the effect of Na_V_ activators, the area under the curve corresponding to 5 min after the addition of veratridine or 30 min after the addition of deltamethrin was used. To quantify the effect of tetracaine (Sigma Aldrich), a two-addition protocol was used, with pre-incubation of tetracaine for 5 min followed by the addition of veratridine (60 μM) (Alomone Labs) to activate Na_v_1.1–1.7 or deltamethrin (150 μM) (Sigma Aldrich) to activate Na_V_1.8.

### FLIPR calcium dye assay

SH-SY5Y human neuroblastoma cells were plated at a density of 50,000 cells per well and MDA-MB-468 breast cancer cells were plated at a density of 10,000 cells per well on black-walled 384-well imaging plates and cultured for 48 h. Fluo-4-AM (Invitrogen) was diluted to 10 μM in PSS from a 10 mM DMSO stock with or without the presence of the quenchers Brilliant Black BN, Ponceau 4R, Allura Red and Amaranth (1 mM). FLIPR Calcium 4 no-wash dye (Molecular Devices) and BD Calcium Assay (BD Biosciences) kits were diluted in physiological salt solution as per manufacturers instructions. Growth media was removed and replaced with 20 μL/well fluo-4-AM or commercial assay kits and cells were incubated in the dark at 37°C for 30 min. The change in fluorescence was measured (excitation 470–495 nm, emission 515–575 nm) every 1 s after addition of compounds using the FLIPR^TETRA^ (Molecular Devices). Raw fluorescence readings were converted to response over baseline using the analysis tool of Screenworks 3.1.1.4 (Molecular Devices) and were expressed relative to the maximum increase in fluorescence of control responses following 5 min incubation of compounds.

### ImageXpress calcium dye assay

MDA-MB-468 breast cancer cells were plated at 30,000 cells per well in 96-well imaging plates (Falcon, 353219) and allowed to attach overnight. Cells were loaded with fura-5F (4 μM, Setareh Biotech) in media for 30 min at 37 ºC. Ca^2+^ imaging was performed in physiological salt solution containing Brilliant Black BN (1 mM). Intracellular Ca^2+^ measurements were recorded using a 10× objective on an ImageXpress Micro automated epifluorescence microscope. Changes in intracellular Ca^2+^ are expressed as the ratio of fluorescence (F340/380) normalized to starting fluorescence.

### Nikon Eclipse TE300 Calcium imaging

MDA-MB-468 breast cancer cells were plated at 20,000 cells per well in 96-well imaging plates (Falcon, 353219) and allowed to attach for 48 h. Cells were loaded with fura-5F (4 μM, Setareh Biotech) in media for 30 min at 37 ºC. Ca^2+^ imaging was performed in physiological salt solution (with 1.8 mM Ca^2+^) containing Ponceau 4R. Intracellular Ca^2+^ measurements were recorded using a 10× objective (Nikon Plan Fluor 10×/0.30) on a Nikon Eclipse TE300 Inverted Microscope. Changes in intracellular Ca^2+^ were recorded using MetaFluor version 7.10.2.240 (Molecular Devices) and are expressed as the ratio of fluorescence (F340/380) normalized to starting fluorescence.

### Whole-cell patch-clamp electrophysiology

Automated whole-cell patch-clamp recordings were performed on HEK293 cells heterologously expressing human Na_V_1.7 (SB Drug Discovery, Glasgow, UK) using a QPatch-16 automated electrophysiology platform (Sophion Bioscience, Ballerup, Denmark) as previously described [9]. The extracellular solution consisted of (in mM): NaCl 70, Choline chloride 70, KCl 4, CaCl_2_ 2, MgCl_2_ 1, HEPES 10 and glucose 10; pH 7.4; osmolarity 305 mOsm. The intracellular solution consisted of (in mM): CsF 140, EGTA/CsOH 1/5, HEPES 10 and NaCl 10; pH 7.3 with CsOH; osmolarity 320 mOsm. Ponceau 4R (1 mM) was diluted in extracellular solution and incubated for 5 min before running the voltage protocol and compared to time-matched buffer controls. Patch clamp experiments were conducted at room temperature.

*I-V* curves were obtained with the membrane potential held at –90 mV and a series of 500 ms step pulses that ranged from –110 to +55 mV in 5-mV increments. Conductance-voltage curves were calculated using the equation G = I/(V–V_rev_), where G is conductance, I is peak current and V_rev_ is the reversal potential. Steady-state fast inactivation was tested using a two-pulse protocol, with 500 ms step pre-pulses (as above) preceding a 10 ms pulse of –20 mV (repetition interval 6000 ms). Both steady-state activation and inactivation curves were fitted using Boltzmann function.

### Data analysis

Data were plotted and analyzed using GraphPad Prism, version 6.0. For concentration-response curves, a four-parameter Hill equation with variable Hill coefficient was fitted to the data. Statistical significance was defined as *P* < 0.05 and was determined by an unpaired t-test assuming equal variance or one-way ANOVA with Dunnett’s post-test as indicated. Data are presented as mean±SEM.

## Results

### Red and violet food dyes have an overlapping absorbance spectra with the emission spectrum of ANG-2

**Fig 2:**
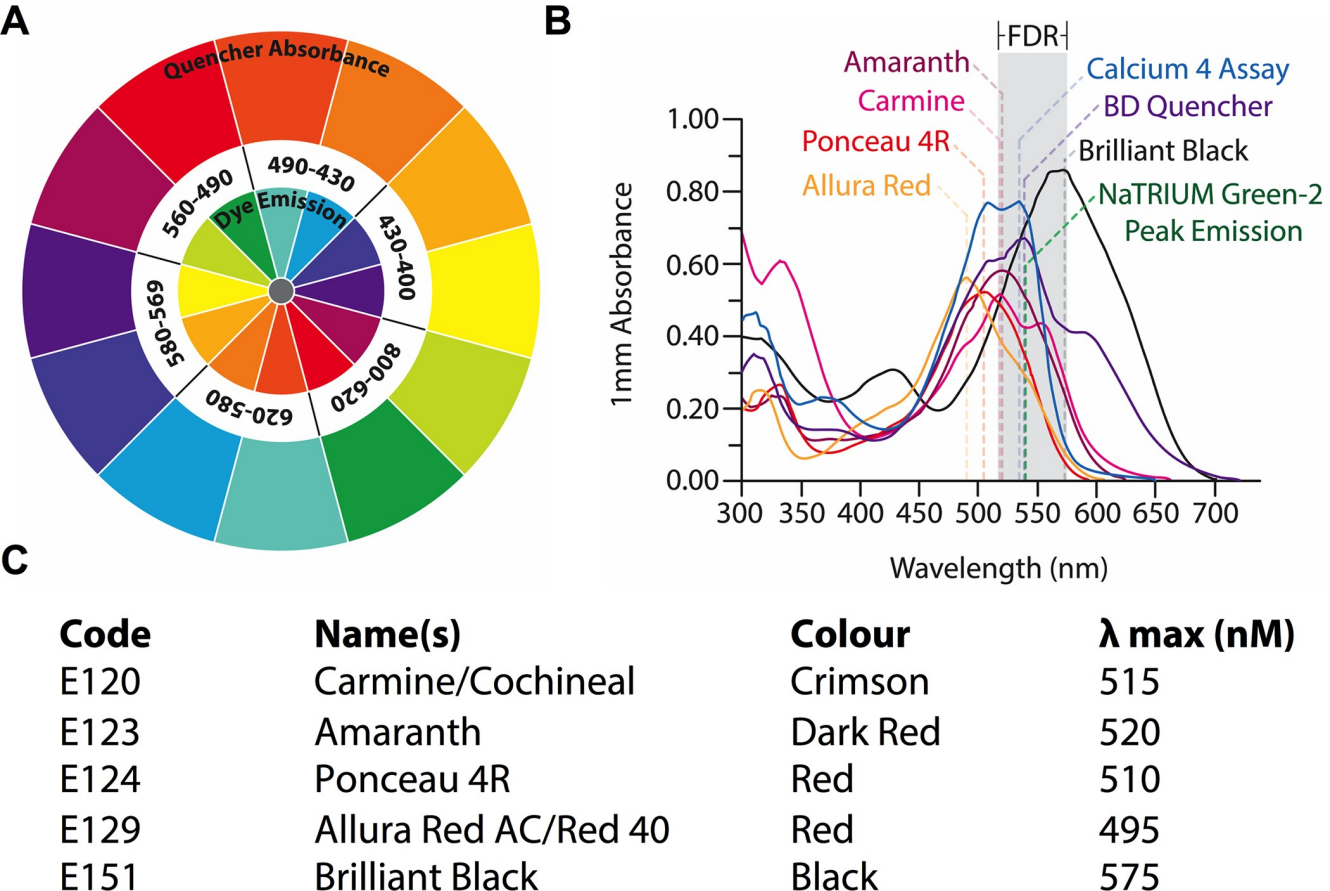
Absorbance spectra of food dyes in the red-violet color range. (A) Schematic diagram of a color wheel illustrating the emission wavelengths (inner circle) and absorption wavelengths (outer circle) of visible light (in nanometers; nm). The inner circle represents the emission color of the indicator dye and the outer circle represents the color of the quencher required to absorb the emission. For example, ANG-2 has a peak emission wavelength of 540 nm (green), therefore it can be expected that red-violet colored dyes will quench the emission (B) Absorbance spectra of Brilliant Black BN, Carmine, Ponceau 4R, Allura Red and Amaranth, Calcium 4 Assay and BD Quencher. Grey shaded area represents the FLIPR^TETRA^ detection range (FDR). (C) International Numbering System for Food Additives (INS) E number, common name(s), color and peak absorption (λ max) of food dyes tested.

ANG-2 has a peak emission at a wavelength of 540 nm, which is in the green visible color range (490–560 nm), so we hypothesized that red and violet colored food dyes would absorb emissions at this wavelength (Fig 2A). We therefore assessed the absorbance spectra of a range of red and violet food dyes, as food dyes are commercially available to purchase and are relatively cheap, making them suitable candidates for use as quenchers in high-throughput assay formats. We also assessed the absorbance spectra of the FLIPR Calcium 4 no-wash dye and BD Calcium Assay for comparison. All of the red and violet food dyes tested, including Brilliant Black BN, Carmine, Ponceau 4R, Allura Red and Amaranth, absorbed at wavelengths that overlapped with the peak emission of ANG-2 (540 nm) and the FLIPR detection range (515–575 nm) (Fig 2B), with varying peak absorbances and ranges (Fig 2C). We therefore hypothesized that these dyes could be used to quench background fluorescence in cells loaded with ANG-2.

### Addition of a red or violet food dye to cells loaded with ANG-2 effectively quenches background fluorescence

**Fig 3:**
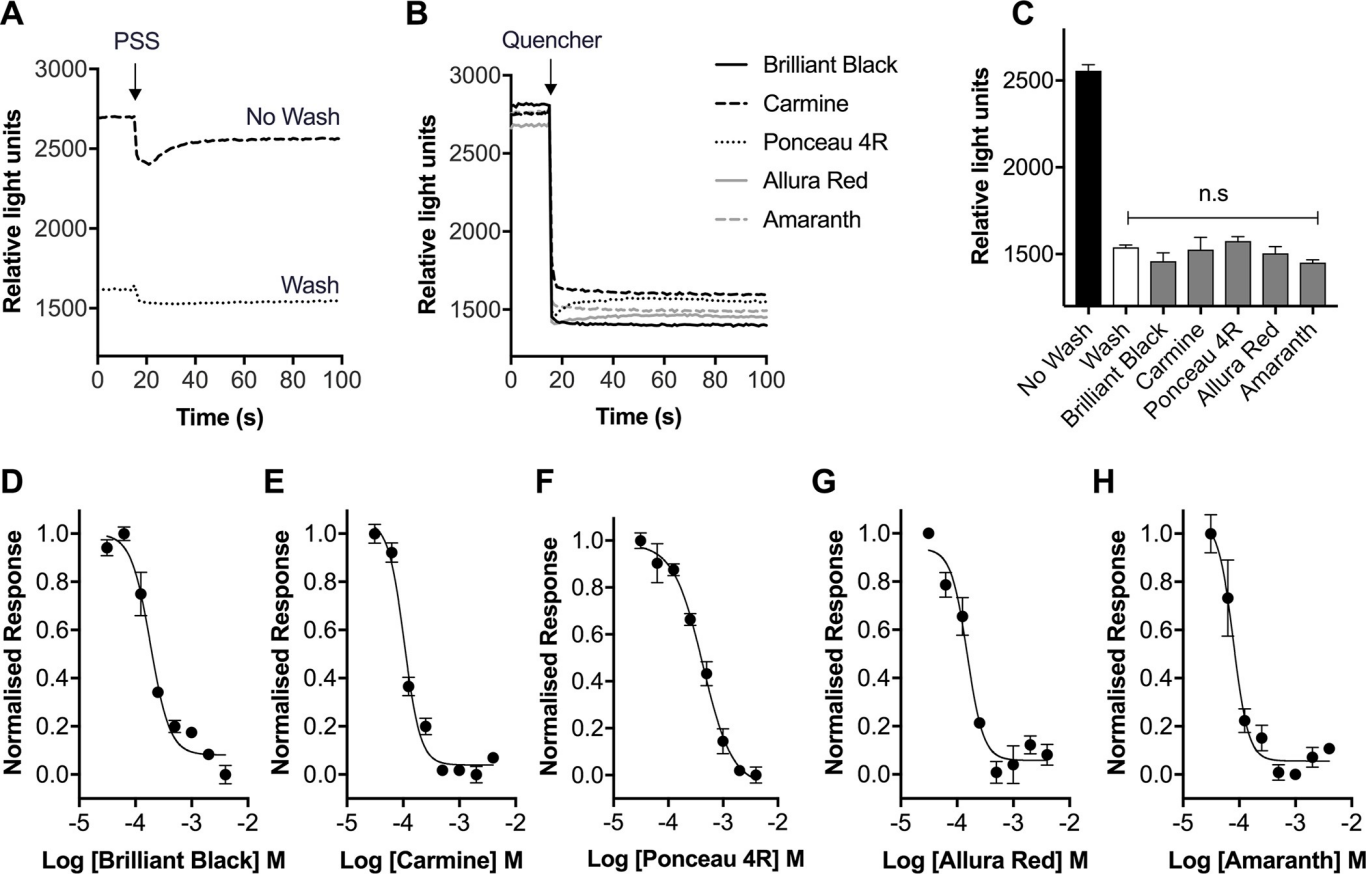
Food dyes in the red-violet color range quench the background fluorescence of the sodium indicator ANG-2. (A) Sample traces of the fluorescence levels of Na_V_1.7-HEK cells with ANG-2 and PSS buffer addition, with and without washing, obtained using the FLIPR^TETRA^. (B) Sample traces of the fluorescence level of Na_V_1.7-HEK cells with ANG-2 following the addition of Brilliant Black BN (1 mM), Carmine (1 mM), Ponceau 4R (1 mM), Allura Red (1 mM) or Amaranth (1 mM) obtained using the FLIPR^TETRA^. (C) The average relative light units corresponding to 60–100 s after addition of quenchers or buffer. Reduction in background fluorescence by Brilliant Black BN (1 mM), Carmine (1 mM), Ponceau 4R (1 mM), Allura Red (1mM) and Amaranth (1 mM) was not significantly different to washing. Statistical significance was determined using one-way ANOVA with Dunnett’s post-test, *P < 0.05 compared to wash. (D-H) Concentration-response curves for the reduction in background fluorescence of Na_V_1.7-HEK cells with ANG-2 by quenchers Brilliant Black BN, Carmine, Ponceau 4R, Allura Red and Amaranth. Data are presented as mean ± SEM from three wells.

Since red and violet food dyes have absorbance spectra that overlap with the emission spectrum of ANG-2, we next assessed their ability to quench background fluorescence in cells loaded with ANG-2 using the FLIPR^TETRA^. Na_V_1.7-HEK cells loaded with ANG-2 have an average background fluorescence of 2557 ± 34 RFU, which is reduced to 1539 ± 44 RFU with washing (Fig 3A). Addition of red or violet food dyes (1 mM) to cells loaded with ANG-2 immediately quenches background fluorescence (Fig 3B) to levels that are not significantly different from washing (Brilliant Black BN, 1459 ± 34 RFU; Carmine, 1526 ± 71 RFU; Ponceau 4R, 1575 ± 26 RFU; Allura Red, 1505 ± 39 RFU; Amaranth 1451 ± 17 RFU; Fig 3C). The ability of red and violet food dyes to quench background fluorescence was concentration dependent (Fig 3D–3H), with the following rank order of potency: Amaranth (IC_50_ 78 μM) > Carmine (IC_50_ 105 μM) > Allura Red (IC_50_ 152 μM) > brillant black (IC_50_ 185 μM) > Ponceau 4R (IC_50_ 427 μM). As all of the food dyes quenched background fluorescence to a similar level, we next assessed their ability to quench background fluorescence in the presence of the Na_V_ channel activator veratridine.

### Pre-incubation of cells with ANG-2 and Ponceau 4R enables a no-wash sodium influx assay without affecting Na_V_ channel function

**Fig 4:**
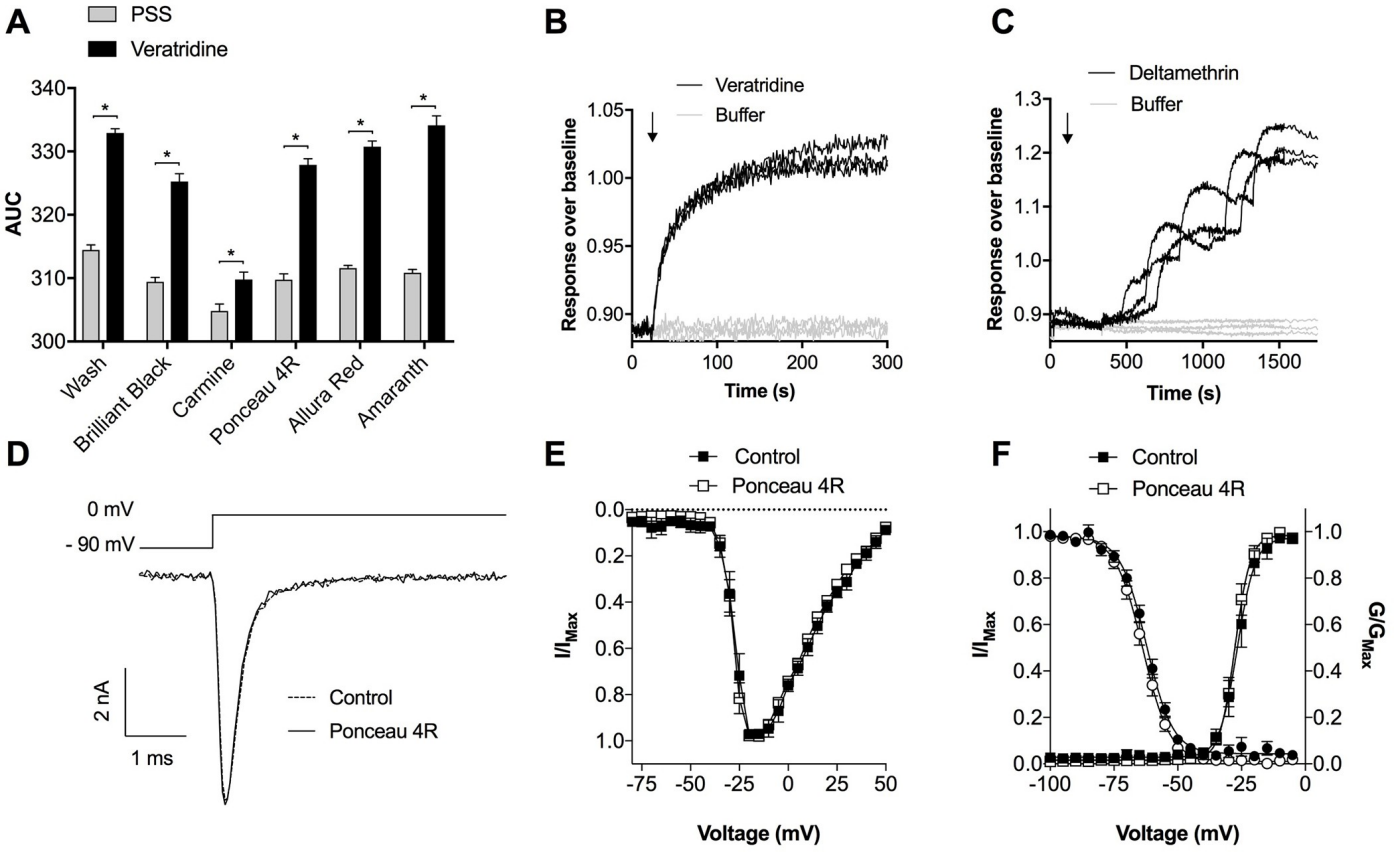
Ponceau 4R quenches background fluorescence of the sodium dye indicator ANG-2 without affecting Na_V_ channel function. (A) Fluorescent response of PSS buffer or veratridine (60 μM) at Na_V_1.7 (quantified by AUC) in the presence of ANG-2 with washing or preincubation with Brilliant Black BN (1 mM), Carmine (1 mM), Ponceau 4R (1 mM), Allura Red (1 mM) or Amaranth (1 mM). Data are presented as mean ± SEM from three wells. Statistical significance was determined using *t*-test, *P < 0.05 compared to buffer control. (B) Sample traces of the fluorescence response obtained using ANG-2 with Ponceau 4R (1 mM) at Na_V_1.7 stimulated with veratridine (60 μM) from three wells. (C) Sample traces of the fluorescence response obtained using ANG-2 with Ponceau 4R (1 mM) at Na_V_1.8 stimulated with deltamethrin (150 μM) from three wells. (D) Representative Na_V_1.7 current trace before (dashed) and after (solid) addition of Ponceau 4R (1 mM) obtained from whole-cell patch clamping. (E) *I-V* curve of Na_V_1.7 with Ponceau 4R (1 mM; white squares) compared to time-matched buffer control (black squares) (F) G-V curve of Na_V_1.7 with Ponceau 4R (1 mM; white squares) compared to time-matched buffer control (black squares). Voltage-dependence of steady-state fast inactivation at Na_V_1.7 with Ponceau 4R (1 mM; white circles) compared to time-matched buffer control (black circles). Data are presented as mean ± SEM, n = 4–7 cells.

Since all of the tested red and violet food dyes effectively quenched background fluorescence, we next assessed which food dye added with ANG-2 produced the best fluorescence signal in response to the non-selective Na_V_ activator veratridine, which is commonly used to activate Na_V_ channels in fluorescence based assays [10]. Addition of veratridine (60 μM) to Na_V_1.7-HEK293 cells loaded with ANG-2 without washing did not result in a fluorescence signal distinguishable from buffer control (S1 Fig), confirming the need for a wash step or quencher in this assay. In Na_V_1.7-HEK293 cells loaded with ANG-2 and washed, veratridine (60 μM) causes an increase in fluorescence, indicative of an increase in the intracellular concentration of Na^+^ in response to Na_V_ channel opening. Addition of veratridine to cells pre-incubated with ANG-2 and a quencher (1 mM) also led to an increase in fluorescence, with responses significantly greater than buffer control addition (Fig 4A). This indicates that all of the food dyes tested quenched background fluorescence sufficiently to detect an increase in intracellular Na^+^ with ANG-2, although Carmine resulted in a comparatively low signal (Fig 4A). This may be because carmine crossed the cell membrane and quenched the intracellular signal. While membrane permeability was not measured, an octonol-water partition test identified carmine as the most likely of the quenchers tested to be membrane permeable (S2 Fig).

As Brilliant Black BN, Ponceau 4R, Allura Red and Amaranth resulted in a similar veratridine-induced fluorescence response, we chose to use Ponceau 4R (1 mM) for all further experiments, as this quencher is the cheapest to purchase in the form of commercially available food dyes (e.g. Queen Pillarbox Red). Sample traces of the fluorescence response obtained using ANG-2 with Ponceau 4R in Na_V_1.7-HEK cells stimulated with veratridine and Na_V_1.8-HEK cells with deltamethrin are shown in Fig 4B and 4C. The high-signal to noise in Fig 4B corresponded to a Z’ factor of 0.60 (n = 18 wells), as calculated using the method described by Zhang et al, confirming the suitability of the no-wash assay for high-through put screening [11].

To ensure Ponceau 4R (as well as the excipients) does not interfere with Na_V_ channel function, we next assessed effects on the electrophysiology parameters of Na_V_1.7 using automated whole-cell patch clamping. Ponceau 4R had no effect on the activation or inactivation kinetics of Na_V_1.7 (Fig 4D) and did not cause a shift in the *I-V* curve or affect the current size (Fig 4E). Ponceau 4R also did not cause a significant shift in the voltage-dependence of activation (V_1/2_ activation: Δ = –0.96 mV) or voltage-dependence of steady-state fast inactivation (V_1/2_ inactivation: Δ = –1.46 mV) at Na_V_1.7 (Fig 4F). This indicates that Ponceau 4R does not directly interact with Na_V_1.7 and is therefore a suitable quencher for Na_V_ activity assays.

**Fig 5:**
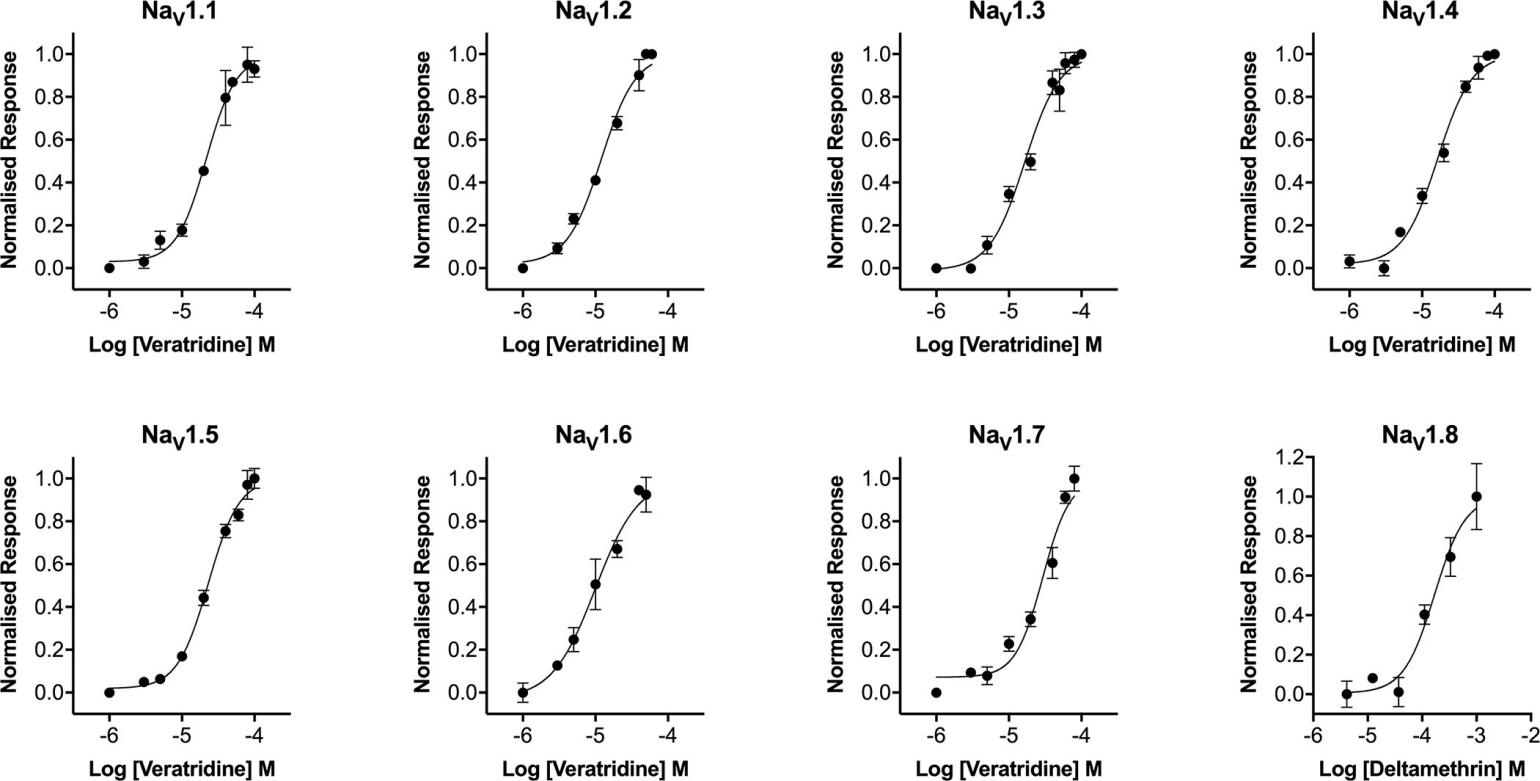
Concentration-response curves for veratridine at hNa_V_1.1–1.7 and deltamethrin at hNa_V_1.8 obtained using a no-wash sodium influx assay using ANG-2 and the quencher Ponceau 4R. Addition of the non-selective Na_V_ channel activator veratridine concentration-dependently increased fluorescence with the following EC_50_’s: Na_V_1.1 21 μM, Na_V_1.2 16 μM, Na_V_1.3 12 μM, Na_V_1.4 16 μM, Na_V_1.5 23 μM, Na_V_1.6 10 μM, Na_V_1.7 29 μM. Addition of the Na_V_ channel activator deltamethrin concentration-dependently increased fluorescence at Na_V_1.8 with an EC_50_ of 169 μM. Data are presented as mean ± SEM from three wells.

**Fig 6:**
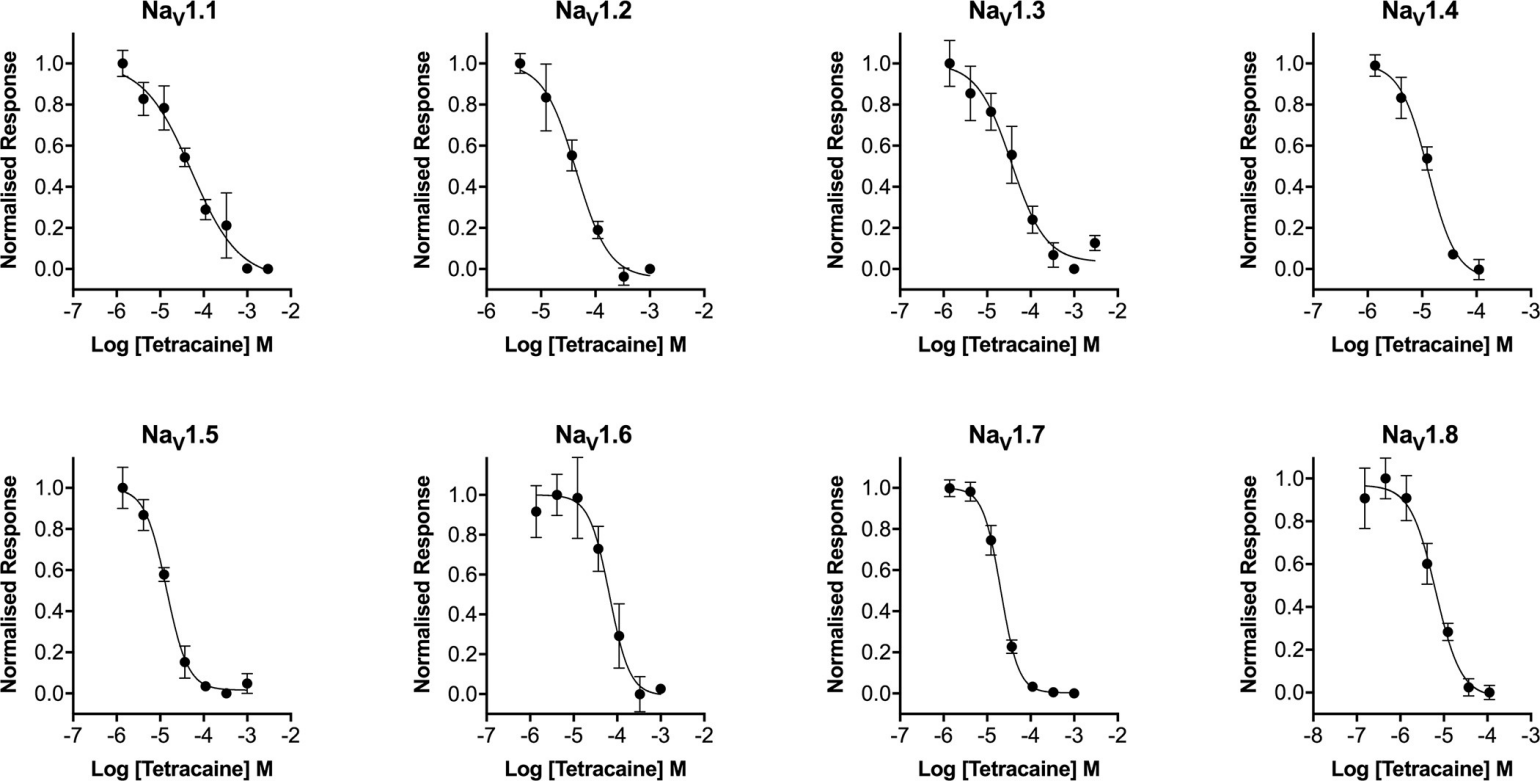
Concentration-response curves for the Na_V_ channel inhibitor tetracaine at hNa_V_1.1–1.8 obtained using a no-wash sodium influx assay ANG-2 and the quencher Ponceau 4R. Preincubation of tetracaine concentration-dependently reduced the fluorescence response caused by addition of veratridine (60 μM; Na_V_1.1–1.7) or deltamethrin (150 μM; Na_V_1.8) with the following IC_50_’s: Na_V_1.1 52 μM, Na_V_1.2 44 μM, Na_V_1.3 36 μM, Na_V_1.4 13 μM, Na_V_1.5 14 μM, Na_V_1.6 66 μM, Na_V_1.7 21 μM, Na_V_1.8 6 μM. Data are presented as mean ± SEM from three wells.

To further validate the pharmacology of Na_V_ responses in the presence of Ponceau 4R, we next assessed concentration-response curves of representative activators and inhibitors at Na_V_1.1–1.8. Consistent with results obtained using membrane potential dyes and patch-clamp electrophysiology, veratridine elicited responses in ANG-2 loaded cells expressing Na_V_1.1–1.7 (Fig 5, Table 1) with EC_50_’s in the range of 10–29 μM, consistent with previously reported values [12]. As veratridine has little effect on Na_V_1.8 in fluorescence-based assays [13], we used deltamethrin to activate Na_V_1.8-HEK cells, which produced a concentration response curve with an EC_50_ of 169 μM (Fig 5, Table 1). ANG-2 with Ponceau 4R also produced concentration-response curves with the Na_V_ channel inhibitor tetracaine, following stimulation with veratridine (Na_V_1.1–1.7) or with deltamethrin (Na_V_1.8), producing IC_50_’s in the range of 6–66 μM (Fig 6, Table 1), which is consistent with closed-state IC_50_’s obtained from patch-clamp electrophysiology [14].

**Table 1:** Potency of veratridine, deltamethrin and tetracaine on Na_V_1.1-Na_V_1.8 determined using the no-wash sodium influx assay.

Na_V_	Veratridine pEC50 (M)	Deltamethrin pEC50 (M)	Tetracaine pIC50 (M)
1.1	-4.66 ± 0.1	N/A	-4.28 ± 0.2
1.2	-4.78 ± 0.1	N/A	-4.35 ± 0.1
1.3	-4.91 ± 0.1	N/A	-4.44 ± 0.1
1.4	-4.79 ± 0.1	N/A	-4.89 ± 0.1
1.5	-4.64 ± 0.1	N/A	-4.86 ± 0.1
1.6	-5.00 ± 0.1	N/A	-4.18 ± 0.1
1.7	-4.53 ± 0.1	N/A	-4.69 ± 0.1
1.8	N/A	-3.77 ± 0.1	-5.18 ± 0.1

### Addition of a red or violet food dye to cells loaded with calcium indicators fluo-4, fura-2 and fura-5F effectively quenches background fluorescence

**Fig 7:**
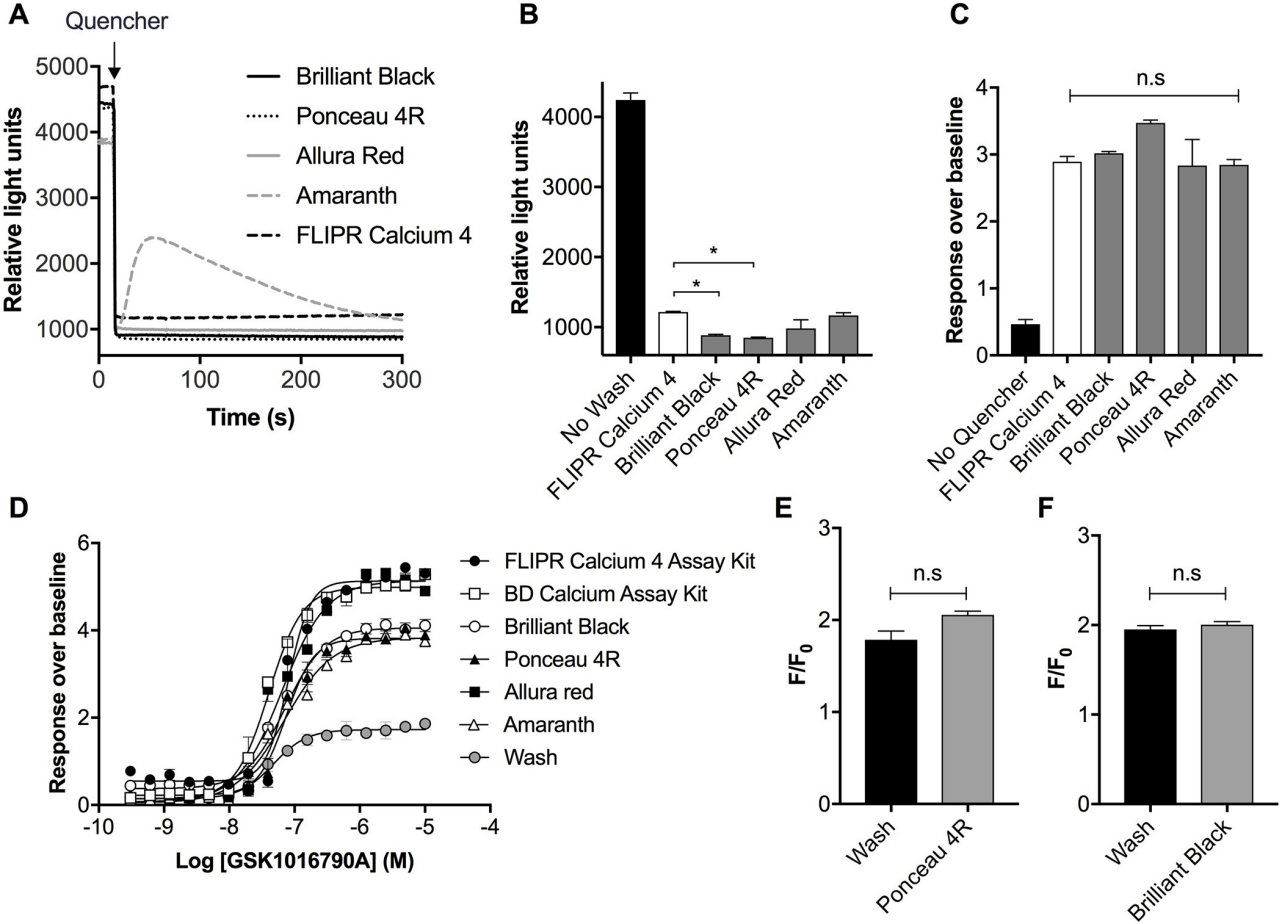
Food dyes in the red-violet color range quench the background fluorescence of the calcium indicators fluo-4, fura-2 and fura-5F. (A) Sample traces of the fluorescence level of SH-SY5Y cells with fluo-4 following the addition of Brilliant Black BN (1 mM), Ponceau 4R (1 mM), Allura Red (1 mM) or Amaranth (1 mM) compared to the FLIPR Calcium 4 Assay Kit obtained using the FLIPR^TETRA^. (B) The average relative light units corresponding to 270–300 s after addition of quenchers or FLIPR Calcium 4 Assay Kit. Statistical significance was determined using one-way ANOVA with Dunnett’s post-test, *P < 0.05 compared to wash. (C) Fluorescent response of KCl (100 mM) on SH-SY5Y cells (quantified by maximum response) in the presence of fluo-4 with washing or preincubation with FLIPR Calcium 4 Kit, Brilliant Black BN (1 mM), Ponceau 4R (1 mM), Allura Red (1 mM) or Amaranth (1 mM). Data are presented as mean ± SEM from three wells. Statistical significance was determined one-way ANOVA with Dunnett’s post-test, *P < 0.05 compared to FLIPR Calcium 4 Assay Kit. (D) Concentration-response curves for TRPV4 channel agonist GSK1016790A on MDA-MB-468 breast cancer cells obtained using FLIPR Calcium 4 Assay Kit, BD Calcium Assay Kit, or Fluo-4 with quenchers (1 mM). Fluorescent response of ATP (100 μM) on MDA-MB-468 breast cancer cells (quantified by maximum response) in the presence of (fura-5F with washing or preincubation with either (E) Ponceau 4R or (F) Brilliant Black BN. Statistical significance was determined using *t*-test, *P < 0.05 compared to wash. Data are presented as mean ± SEM from three wells.

As the absorbance spectra of red and violet food dyes also overlap with the peak emission wavelength of the commonly used calcium indicator fluo-4 (510 nM), we next assessed their ability to quench background fluorescence compared to commercial no-wash calcium assay kits. SH-SY5Y cells loaded with fluo-4 have an average background fluorescence of 4243 ± 100 RFU, which is reduced to 1217 ± 9 RFU with the FLIPR Calcium 4 no-wash dye (Fig 7A and 7B). Addition of red or violet food dyes (1 mM) to cells loaded with fluo-4 immediately quenched background fluorescence (Fig 7A) to levels that were either not significantly different or significantly lower than the commercial kit (Brilliant Black BN, 885 ± 12 RFU; Ponceau 4R, 848 ± 11 RFU; Allura Red, 980 ± 126 RFU; Amaranth 1166 ± 40 RFU; Fig 7B). In response to stimulation with KCl (100 mM), the fluorescent response over baseline in SH-SY5Y cells loaded with the FLIPR Calcium 4 no-wash dye was not significantly different to fluo-4 pre-incubated with Brilliant Black BN, Ponceau 4R, Allura Red or Amaranth (Fig 7C). Addition of the TRPV4 agonist GSK1016790A in MDA-MB-468 breast cancer cells loaded with FLIPR Calcium 4 no-wash dye, BD Calcium Assay Kit, or fluo-4 with quenchers or washing led to a concentration-dependent increase in intracellular calcium (Fig 7D). While the maximum response size differed between the commercial kits and quenchers, the EC_50_’s obtained were similar (EC_50_ GSK1016790A: FLIPR Calcium 4 no-wash dye 80 nM, BD Calcium Assay Kit 40 nM, Brilliant Black BN 72 nM, Ponceau 4R 74 nM, Allura Red 64 nM, Amaranth 70 nM, wash 47 nM). Finally, we also tested whether food dyes could also be used to quench the background fluorescence of the calcium indicators fura-2 and fura-5F, which have peak fluorescence emission wavelengths of 510 nm and 506 nm, respectively. Indeed, in MDA-MB-468 breast cancer cells loaded with fura-2 and fura-5F, the maximum response to stimulation with ATP (100 μM) did not significantly differ between washing or pre-incubation with Brilliant Black BN (Fig 7E and 7F).

## Discussion

Here we describe the development of a high-throughput fluorescent no-wash sodium influx assay using the sodium dye indicator ANG-2 and commercially available food dyes as quenchers. While the experimental protocol for ANG-2 outlines the need for a wash step, the addition of commercially available food dyes with ANG-2 negates the need for this step. In our hands, we found the wash step difficult to implement in 384-well format, as HEK293 cells stably expressing Na_V_1.1–1.8 have low adherence, and often detach after the wash step despite the use of surfaces that improve cell attachment such as CellBIND and Poly-D-lysine. While all of the red-violet food dyes tested effectively quenched background fluorescence of ANG-2, we chose to use Ponceau 4R in a non-analytical standard form (as the food coloring Pillar Box Red (Queen)), as it is inexpensive to purchase and thus economical for use as a quencher in high-throughput assay formats. Interestingly, out of all the food dyes tested, carmine had the poorest signal-to-noise ratio following addition of veratridine. (Fig 4A) This could be a result of carmine being membrane permeable (S2 Fig) and quenching the intracellular ANG-2 fluorescent signal. It should be noted that membrane permeability was not tested for the quenchers described here, and that incubation times beyond 30 min may result in increased levels of intracellular quenching and a loss of signal.

For a quencher to be suitable for use in fluorescence-based assays, it must not directly act on or alter the pharmacology of the target of interest, it must be membrane impermeable and it must absorb in the wavelengths that overlap with the emission spectra of the ion indicator dye. Interestingly, the absorbance spectra of all the quenchers (both food dyes and commercial) overlapped with the emission and excitation wavelengths of the sodium and calcium indicator dyes, however this did not reduce the fluorescent signal when compared to the wash assay in our hands.

Ponceau 4R is not known to act on any ion channels or G protein-coupled receptors and is foodsafe. Our results support this, as Ponceau 4R did not alter the electrophysiology parameters of Na_V_1.7 channels. In addition, potency values obtained for veratridine and tetracaine with Ponceau 4R were consistent with values obtained using other methods [12, 14], indicating that Ponceau 4R does not alter the pharmacology of Na_V_ channels. This therefore demonstrates that Ponceau 4R is suitable for use as a quencher in Na_V_ channel assays, although the effects of Brilliant Black BN, Carmine, Allura Red and Amaranth on Na_V_ channels remains to be confirmed. It should be noted that an additional advantage of using quenchers in a non-kit format is that pharmacological effects on the target of interest can be directly assessed. In contrast, the components of commercial no-wash kits are typically undisclosed, which can lead to unintentional assay artifacts that may be difficult to determine and eliminate.

We also provide a proof of concept that food dyes can be used as quenchers with other ion indicators dyes, including the calcium indicators fluo-4, fura-2 and fura-5F. While this has previously been shown for other food dyes, including Brilliant Black BN and Allura Red [5–7], this is the first publication to systematically compare the spectra and quenching activity of a range of different food dyes alongside commercial quenchers. While many commercial no-wash calcium assay kits are available, these generally contain undisclosed or proprietary quenchers, and accordingly are very expensive. The ability to replace commercial no-wash assay kits with a food dye quencher will reduce assay costs, allow more flexibility over the assay parameters, and enable individual testing of quenchers to ensure they do not interact with the assay target. The latter is important to check, as it has recently been reported that Brilliant Black BN is an allosteric modulator of adenosine receptors [15].

In summary, we have developed a no-wash fluorescent sodium influx assay with a signal-to-noise window that is suitable for both screening and pharmacological characterization of Na_V_ channel activators and inhibitors. We have also identified several food dyes with peak absorbance in the wavelength range 495–575 nm that may be used as cheaper alternatives to commercial no-wash ion indicator kits.

## Supporting information

S1 Fig The ANG-2 fluorescence assay requires a wash step to generate a functional signal-to-noise ratio. Addition of veratridine (60 μM) to Na_V_1.7-HEK293 cells loaded with ANG-2 without washing did not result in a fluorescence signal distinguishable from buffer control. Three response over baseline (normalized to negative control) traces are displayed per condition.
(PDF)

S2 Fig Octanol-water partition of quencher dyes. Quenchers were added to 1:1 octanol/water volume to a 1mM concentration. Tubes were agitated and allowed to settle overnight. Allura red and Carmine partitioned into the octanol phase (most notably in carmine), suggesting that the two quenchers may be membrane permeable.
(PDF)
