# Supplementary material for: Development of a high-throughput fluorescent no-wash sodium influx assay
Source: PLoS One. 2019 Mar 11;14(3):e0213751. doi: 10.1371/journal.pone.0213751 (PMC6411159; doi:10.1371/journal.pone.0213751)
Supplement: S1 Fig — Addition of veratridine (60 μM) to NaV1.7-HEK293 cells loaded with ANG-2 without washing did not result in a fluorescence signal distinguishable from buffer control. Three response over baseline (normalized to negative control) traces are displayed per condition. (PDF) [file pone.0213751.s001.pdf]

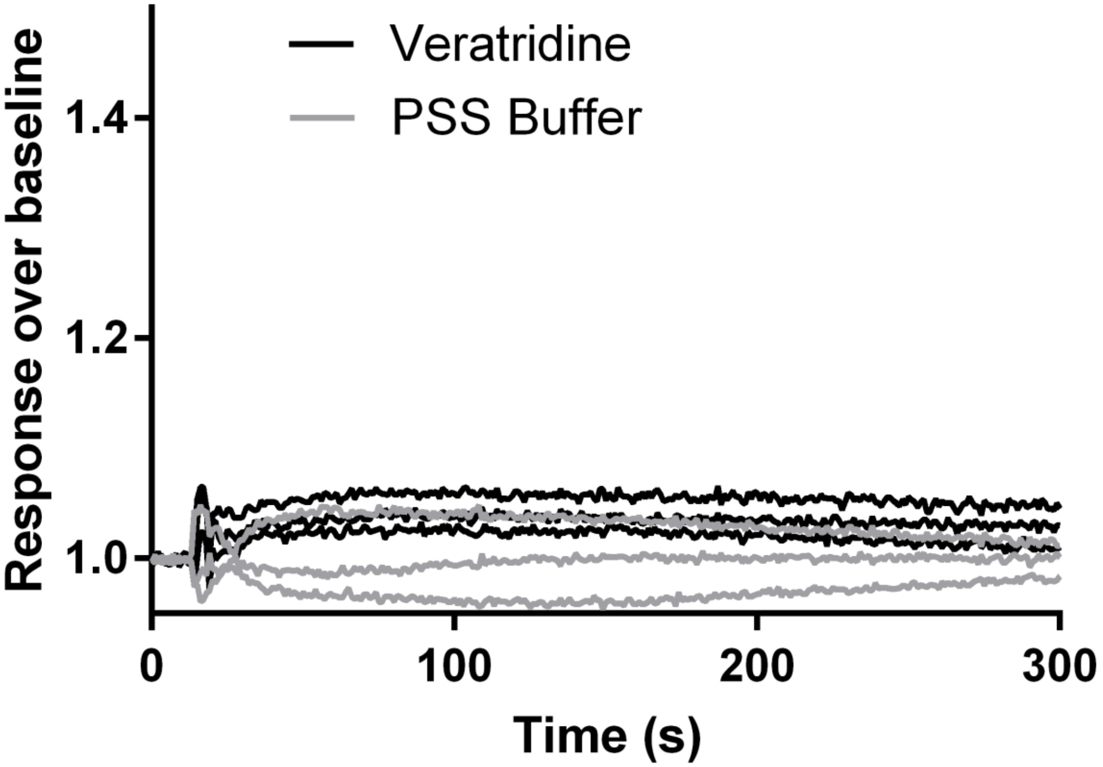

S1 Fig. The ANG-2 fluorescence assay requires a wash step to generate a functional signal-to-noise ratio. Addition of veratridine (60 μM) to $Na_V1.7$-HEK293 cells loaded with ANG-2 without washing did not result in a fluorescence signal distinguishable from buffer control. Three response over baseline (normalized to negative control) traces are displayed per condition.
