# Supplementary material for: Development of a high-throughput fluorescent no-wash sodium influx assay
Source: PLoS One. 2019 Mar 11;14(3):e0213751. doi: 10.1371/journal.pone.0213751 (PMC6411159; doi:10.1371/journal.pone.0213751)
Supplement: S2 Fig — Quenchers were added to 1:1 octanol/water volume to a 1mM concentration. Tubes were agitated and allowed to settle overnight. Allura red and Carmine partitioned into the octanol phase (most notably in carmine), suggesting that the two quenchers may be membrane permeable. (PDF) [file pone.0213751.s002.pdf]

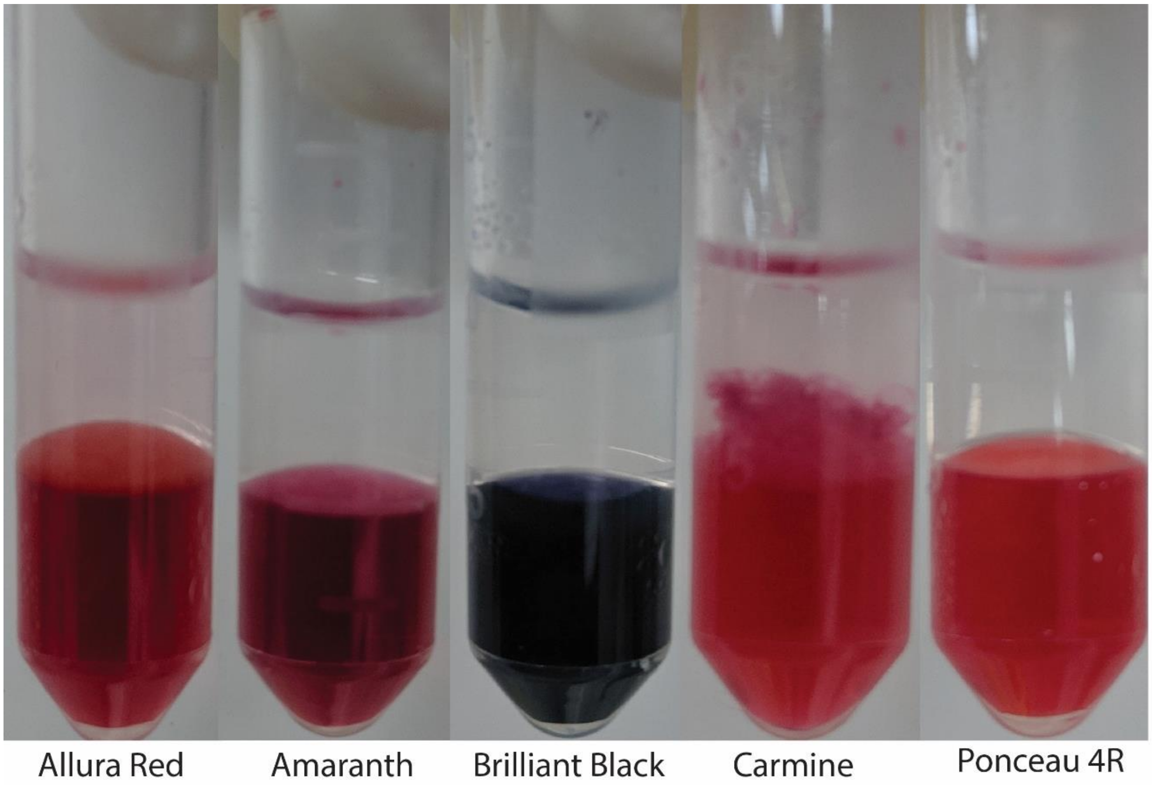

S2 Fig. Octanol-water partition of quencher dyes. Quenchers were added to 1:1 octanol/water volume to a 1mM concentration. Tubes were agitated and allowed to settle overnight. Allura red and Carmine partitioned into the octanol phase (most notably in carmine), suggesting that the two quenchers may be membrane permeable.
